# Empyema of the Maxillary Sinus

**Published:** 1893-02

**Authors:** F. L. Stillman

**Affiliations:** Columbus, O.


// THE DENTAL REGISTER.

VOL. XLVII.] FEBRUARY, 1893. [No. 2.

### COMMUNICATIONS.

### Empyema of the Maxillary Sinus.

BY F. L. STILLMAN, D.D.S., COLUMBUS, O.

Read before the Ohio State Dental Society, December, 1892.

To comprehend certain phenomena in connection with empyema of the maxillary sinus, and in order to make satisfactory differential diagnoses, it is necessary to remember the structure and relations of the maxillary sinus, and of the other closed cavities whose ducts empty into the nasal fossæ.

The maxillary sinus, or Antrum of Highmore, is a pyramidal cavity hollowed out of the superior maxilla. Its upper portion is in relation with the floor of the orbit; in front it is bounded by the anterior wall of the superior maxillary bone; internally it is bounded by the outer wall of the nasal fossa, and below by the alveolar process of the upper jaw. It communicates with the nose by the semi-lunar opening about half an inch long, which is called the ostium maxillare. This opening is situated under and covered by the middle turbinated body. As with other sinuses of the nose, the duct is not situated at the lower portion of the cavity. It will thus be seen that the natural duct will not facilitate thorough drainage in case of collections of purulent matter, while the patient is in the erect position. The interior of the cavity is lined with a continuation of the mucous membrane of the nose, and exhibits similar features. The superficial layer of mucous membrane is rather thin, and is inclined to assume a fold-like arrangement, while the deep layer is substantially the periosteum of the underlying bone.

The other sinuses which communicate with the nose and are liable to similar purulent inflammations, are the frontal, the ethmoidal and the sphenoidal. The frontal sinuses are two in number, and lie between the two plates of the frontal bone. Their ducts emerge on the respective sides of the nasal fossæ, just in front of the ostium maxillare. The ethmoidal cells are situated between the orbits, and are divided into two groups on each side—anterior and posterior. The anterior ethmoid cells communicate with the nasal cavity by a duct which empties into the middle meatus, and the posterior by a duct which communicates with the superior meatus. The sphenoidal cells are situated in the body of the sphenoid bone, and empty by a duct in the superior meatus at the back and upper part of the nose.

In health the secretion of the mucous membrane of the maxillary sinus is just sufficient in amount to moisten and lubricate the membrane. So in health the cavity is simply an air space in the superior maxillary bone. A gradual evaporation of the secretion takes place through the ostium maxillare. Bosworth* thinks that inflammation of the cavity rarely takes place by the advance of inflammation in the nose through a continuity of mucous membrane.

Inflammation, both acute and chronic, of the nasal mucous membrane may, however, be a cause of empyema of the antrum. The turgescence of the tissues in the region of the hiatus, under the middle turbinate, may cause blocking up of that channel, and thus prevent free exit of the discharges. An accumulation then takes place, and gradually the phenomena of pus formation take place. There is another factor in the production of empyema of the maxillary sinus, which interests you, gentlemen, more particularly, and that is the close relationship of the teeth and alveolar process, and the often causal influence of morbid conditions thereby offered.

The roots of the first and second upper molars generally project into, and sometimes pierce, the floor of the sinus. Caries of the tooth or alveolus would thus readily cause a morbid condition

*Diseases of the nose and throat—page 466.

in the floor of the antrum, which could readily produce the disease under consideration.

It could thus be produced even if the ostium maxillare were patent—but if, in addition to the periosteal irritation proceeding from the mouth, the ostium should be more or less occluded by polypi, inflammatory swelling, or other causes, the chances are still more favorable for its development.

It is a mooted question whether the disease is most often caused by diseases of the teeth, or by intra-nasal disease. Lennox-Browne* thinks that it is more often of dental origin, while Bosworth† thinks it is oftener due to intra-nasal causes. The question has never been satisfactorily decided. The reason different observers vary in their statements is probably because of the preponderance of their surgical work being in different fields—thus most of the cases consulting dental surgeons will be due to dental disease, and most of the cases due to intra-nasal troubles will consult the throat surgeon.

As the causes are generally slowly operating, so will the approach of the disease be more or less insidious and chronic. The mucous membrane becomes swollen until it is many times thicker than normal. The turgidity of the tissues will cause an escape of leucocytes, and an abundant pus will be poured forth. The mucous membrane will be thrown into thick folds, to which the more solid products of inflammation will adhere. Polypi may form, which will still further complicate the case and protract its treatment.

In cases of extreme chronicity the inflamed periosteum may form new bone products, which may assume the form of stalagmites, or may form partitions or bridges across the cavity.

In cases due to affections of the teeth patients will complain of neuralgic pains in the jaw, which are intensified by tapping the faulty teeth. Coincidently, neuralgia over the affected cheek will be complained of, and will be more marked where the nerves are superficially placed, or where they make their exits from bony foraminæ. There will be swelling of the cheek, which is

*Locus cit.

†Ibid—page 467, 468.

sometimes reddened. At the same time the patient will have a fetid discharge from the nose, which is found to be from one side only. Owing to the position of the ostium maxillare, the flow of purulent matter is increased by inclination of the head forward and toward the sound side.

Differential diagnosis between the affection under consideration and affection of the other closed sinuses is rather important, and, with a little attention to details a mistake should be impossible. The use of the nasal speculum and reflected light assist very much in making a positive diagnosis, and are important adjuncts in the treatment.

A purulent discharge from the ethmoid cells is more often bi-lateral, and there is more or less pain and feeling of tension at the root of the nose. By reflected light the pus is seen to issue from under the middle turbinates on each side, and also from under the superior turbinates, if the posterior ethmoid cells are involved—which is generally the case. The flow is increased most by inclination of the head forward.

Empyema of the frontal sinuses is accompanied by pain in the frontal region and a discharge under the middle turbinates. It is also, generally, a bi-lateral disease.

Empyema of the sphenoid sinus is accompanied by pain in the back and top of the head, and by a purulent discharge which is seen to issue into the superior meatus of the nose when nasal speculum and reflected light are used.

Thus, briefly, have been recounted the differential diagnoses between empyema of the maxillary sinus and the diseases with which it might most readily be confounded. Other diseases may occasionally lead us to suspect the disease under consideration, but intra-nasal examination should readily clear up the diagnosis.

There is one other mode of examination which gives positive indications when pus is absent from the antrum, and also—providing there is no great anomaly in the size of the antra, demonstrates its presence.

I refer to trans-illumination of the maxillary sinus. Stated briefly, it consists in illuminating the face in a dark room by means of a light placed in the mouth. The best means of illumination is a small electric lamp of from two to four-candle power placed in the mouth, well up toward the roof, and the lips closed on the stem beyond the lamp. If the antra are healthy, the face will be illuminated in the following manner: the translucent glow is marked over the nose, cheeks, and upper lip. The tissues are not so brilliantly illuminated over the molar bones, but a narrow streak of translucency is observed under the lower eye-lids. The pupils of the eyes were also seen to be illuminated if they are examined in the line of vision. Now in case the antrum is diseased, the degree of opacity will depend upon the amount of adventitious elements in its cavity. If there is much thickening of the mucous membrane, or a watery fluid in the antrum, there will be some diminution of the translucency; but if the antrum contains pus, the corresponding side of the cheek will be in deep shadow.

This method of examination may discover unsuspected cases of blocked up antra.

A patient is rather fortunate if the empyema from which he suffers originates from a tooth, for the removal of the offending object makes possible the employment of the best method of treatment. This is true because the opening through the tooth socket offers the best drainage possible. Besides, if the artificial opening for treatment is in this location, it is under ready inspection.

Owing to the inspissation of the products of inflammation, a thorough and methodical "flushing out" of the cavity of the antrum is called for. If the opening through the tooth socket is not large enough, it can be enlarged by a suitable dental drill. The cavity of the antrum can be cleansed from the tooth socket by means of a syringe with a suitably shaped extremity. But more satisfactory work can be done by means of a fountain syringe or similar apparatus. The head is inclined forward, and the fluid allowed to flow from the nose into a suitable receptacle. The amount of fluid used in "flushing out" the diseased antra will depend on circumstances; it will generally be about a quart. A good rule to follow is to keep up the flow until floculi cease to come away. Many different solutions are recommended, such as peroxide of hydrogen—carbolized water, solution of chloride or sulphate of zinc, etc. For ordinary treatment I prefer to cleanse out the antrum with a solution of bicarbonate of soda (one drachm to oz.) ; when the escaping fluid begins to come away clear, a solution of bichloride of mercury (1-5000) is substituted, and is allowed to run a little longer. All fluids used should be milk warm in temperature. If there are no complications, most cases will get well under this treatment in from three to seven weeks. Sometimes in the latter stages resolution will be hastened by a mild astringent, such as sulphate of zinc (2—5 grains to the ounce.) The main thing to bear in mind is to use cleansing, antiseptic and unirritating solutions, and to use sufficient of them to thoroughly remove any detritus that may cling to the folds of swollen membrane. The "flushing out" process should take place often enough to prevent the retention of fetid pus. It will be necessary to keep the opening through the alveolus patent. This can be done in various ingenious ways. Common methods are, a silver tube properly fashioned and bent, with an enlarged plate or shoulder on its oral extremity to prevent its slipping into the antrum. Hard rubber fashioned in proper manner, may also be used. I generally prefer a closely wound spiral of silver wire. It adapts itself to the shape of the fistulous opening, is easily introduced, produces no irritation, and is easily and quickly made.

Chiari* considers that the best method of treatment is tamponing with iodoform gauze. The tampon must be introduced, if possible, by the opening through the alveolus.

Other matters in the way of minutiæ will suggest themselves to those treating this disease, and would take up too much space were they elaborated here.

If an empyema is discovered, and there is not sufficient disease of the first or second molar tooth to justify its removal, it will be necessary to make an opening in some other place to admit of drainage, and to serve as an opening through which the diseased cavity can be properly treated.

This is necessary, because it is all but impossible to catheter-

*Chiari—Wien.—Prager Med. Woch., 1892, Nos. 22, 23, 24.

ize the antrum through the natural opening under the middle turbinate. Furthermore the sacrifice of a sound tooth to gain access to the cavity, is never justified.

The point of election for the puncture is in the inferior meatus, about half an inch posterior to the nasal spine. The bony partition is very thin in this location, and it gives better drainage than when made in the middle meatus. The opening can be made with a straight or slightly curved trocar. This opening may be made to serve the purpose of an exploratory puncture simply, or it can be employed, rarely, to carry out the treatment.

Another site for the artificial opening is in the canine fossa. The mucous membrane is dissected up, and the bony wall is drilled or chiseled through. The latter is undoubtedly the best location for the opening, when the disease seems to be kept up by intra-antral conditions that can only be removed by local surgical interference. The opening should be made large enough to admit the little finger, and large enough to admit of inspection of the cavity of the antrum by means of reflected light, or the electric search light. Then if the discharge is found to be kept up by granulations, or by enormously swollen mucous membrane, this can be thoroughly curetted, and an improvement confidently awaited. Spicula of bone can also be broken and smoothed down, and polypoid degenerations removed. This operation is advocated by W. Robertson, M.D.,* of New Castle-on-Tyne. He makes the opening flush with the floor of the antrum. If there is severe hemorrhage after the curetting, he advises tamponing until it ceases. He then places a lead spigot in the artificial opening, and allows it to remain until the condition of the antrum is such that the opening can be safely allowed to heal.

One thing I consider rather important, no matter what method of treatment is adopted, and that is, to prevent as much as possible swelling of the mucous membrane of the nose during the course of the disease. In simple cases the use of an albolene and menthol, or albolene and eucalyptol spray after the employment

*Journal of Laryngology, Rhinology and Otology, May 1892.

of the douche, will fulfil all indications. If the hiatus semilunaris is encroached upon by any hypertrophy or neoplasm in its neighborhood, this hindrance to natural drainage should be remedied by appropriate treatment.

---

DISCUSSION, BY M. H. FLETCHER, D.D.S., CINCINNATI, O.

---

MR. PRESIDENT:—In listening to the able paper just presented, I find the essayist has gone over the anatomy, etiology, physiology, etc., very much the same as we find it in all text-books, and the point I wish to speak of this morning is that part of the subject which pertains especially to our profession, viz: "The Relation of the Disease of the Maxillary Sinus as connected with the Teeth. This disease is not one of frequent occurrence, and when it does occur is very seldom caused by a diseased tooth.

For the purpose of establishing negative evidence in this line I have computed from my own records the statistics pertaining to the number of pulpless teeth treated in my office; but before giving these statistics I wish to make some quotations from recent authors in surgery, which seem to indicate that this trouble is attributed more largely to diseased teeth than it should be. My first quotation will be from the *American System of Surgery*, edited by Drs. Keen and White, and published within the last year. In their article on this disease they say: "The most common affection of the antrum is inflammation with subsequent abscesses. This may be the result of a propagation of the inflammatory lesion from the nose, or not unfrequently of disease following up the roots of the teeth, which sometimes project a little distance into this cavity. When we remember the ease of communication from one cavity to another (meaning from the nose to the antrum) the wonder is that abscess does not occur more frequently than is the case. Not a few cases have followed attacks of the grip, in which the nasal symptoms were especially severe. The presence of polypi seems to be, sometimes, the exciting cause. The second form appears to be an extension by an alveolar periostitis, which is the result of dental caries."

It seems from this that the pathology of dental abscess is not thoroughly comprehended by a great many writers, for personally I have not seen nor can I readily conceive of the antrum frequently becoming diseased from an attack of periostitis or better periodontitis.

"The third form may arise from purely traumatic causes."

As to the treatment, these same authors say: "The treatment of inflammation previous to the formation of an abscess may consist in leeching, and hot applications with appropriate general measures. When pus has already accumulated, a free opening for its evacuation is essential. If the disease has extended upward from a tooth, the old method of the removal of the tooth and the perforation of the antrum at its base will be proper; but when the teeth are sound it is a pity to sacrifice one of them for this purpose, and it is preferable to perforate the antrum at the point of election, which is *above the point of the root of the second bicuspid*, about an inch above the border of the gum. The mucous membrane of the buccal fold may be incised at this point, and the thin wall of bone perforated with a trocar or delicate gouge with the exertion of very little force."

In the last edition of Bryant's Surgery, under Treatment of this Disease, we read that "Suppuration of this cavity is often due, doubtless, to the extension of inflammation from the teeth."

The facts as stated from these two works seem to give the idea expressed by all the authors that have come under my knowledge, on the subject, and it would seem that they copy one from another on this disease, without practical observation or experience. This I deem to be a great defect in our text-books, on numbers of subjects, many men copying from previous writers or writing *for* experience, instead of *from* experience, and in this way we get our minds filled with statements which are not always founded on facts.

As another instance of lack of observation, I quote again from the *American System of Surgery*, in regard to treatment of Alveolar Abscess, or dental abscess, as I deem it should be called. On page 657 of this work we read as follows: "Abscesses of the gums and jaws, or gum-boil, is a very common affection, and is due to irritation from decayed teeth. Sometimes it is quite superficial and can be cured by puncture, but at other times when connected with the diseased root of a tooth it is much deeper and is accompanied by considerable swelling of the face and severe pain. In such cases exit may be found for the pus along the surface of the root, and temporary relief be afforded."

This statement to one who is familiar with treating dental abscess seems to show a lack of a thorough comprehension of the pathology of the case.

I wish now to present my own statistics as to the treatment of pulpless teeth in a sort of negative evidence, as stated, to the end that very few diseased antrums come primarily from diseased teeth. I have taken from my records statistics on this subject running back six years, and could double the number by going over the six previous years through which careful record has been kept. The statistics for the last six years show the treatment of 482 cases, and as can be seen by referring to the diagram which I hold in my hand the upper teeth are much more liable to this trouble than those in the under jaw—there being, in the upper jaw, 352 cases, and in the lower jaw 177 cases; thus showing a large preponderance of the upper teeth which are in proximity to the antrum of Highmore. Those from which there is a probability of having disease in this cavity are the canine, the bicuspids, and the first and second molars. Of these teeth I have treated: on the left side, 129 cases, and on the right side 98 cases, making in all 227 cases, and out of this number, or twice this number which my records would show, I have yet to see but one case coming from a diseased tooth; and this case, strange to say, has presented itself since receiving a synopsis of Dr. Stillman's paper.

The case is as follows: Mrs. C., aged 47, presented herself one month ago for the purpose of having her teeth extracted, preparatory to the insertion of a full denture. Not wishing to extract the teeth myself, I sent her away for that purpose. On presenting herself last Saturday for the examination of the mouth, she complained of pain on the right side of the face, and some soreness of the gums about the socket of the second molar tooth.

A close examination showed an inflammatory condition of the mucous membrane in that locality and slight tenderness on pressure. Seeing the socket from which the tooth had been taken was still patulous I proceeded to examine with a sound. Finding the sound progressed further and further through the socket, I soon came to the knowledge that it had entered the antrum and found it was followed by the discharge of a quantity of exceedingly offensive pus. This, of course, was sufficient evidence of antral disease. We then proceeded to extract the pus to thorough cleanliness of the antrum as far as possible. In so doing, it seemed to me that there must have been a discharge of something like an ounce of pus. The history as given by the patient is that, beginning with July last, she was sick for weeks, with a disease of that side of the face and copious discharge of pus about the back teeth ; this having determined her, to some degree, to have the teeth extracted and have an artificial denture. I did not get this part of her history until she presented herself last Saturday, or the trouble with the antrum might have been discovered earlier.

The only other case of antral trouble that has come in my practice as connected with the teeth, is that of a boy of seventeen, of strumous diathesis, whose antral trouble was caused by undue exposure during our Cincinnati flood, in 1883, he having been in the water for a considerable length of time, removing furniture from a residence. This case involved all the teeth on the diseased side, but it seemed to show that the disease of the teeth was the result of the disease of the antrum rather than the reverse. The first upper molar tooth on the diseased side had been treated a year before for the death of the pulp, and the roots filled. This tooth partook less of the severe inflammation than any other on that side. The trouble seemed to have located and culminated, so far as the teeth were concerned, about the root of the lateral incisors. Now, with these statistics, and those which many of you might give from your records, I believe it could be conclusively established that more teeth are diseased from trouble in the antrum than in cases where the antrum is diseased from any affection of the teeth.

As to the point of perforation of the antrum, the *American System of Surgery* states as quoted: "If not desirable to extract a molar tooth a point of election would be above the point of the root of the second bicuspid, about an inch above the border of the gum."

This would seem to be a bad place, from the fact that a perforation above the point of the root of any tooth would destroy the nerve and blood supply to the pulp of that tooth.

I hold in my hand a superior maxillary bone perforated into the antrum through the socket of the first molar tooth, and also at a point which I deem much better than above the bicuspid, which point is between the roots of the second bicuspid and the first molar tooth, thus avoiding the interference with the blood and nerve supply of any tooth, and at the same time coming nearer the floor of the antrum, thereby producing better drainage. The perforation can be made first with a small fissure drill by first dissecting the soft tissues down to the bone, then using the drill to make the opening. Through this opening an examination can be made with a sound, thereby finding how near the floor you may be, and the enlargement made in any direction desired and to any extent necessary, without in any way interfering with the future life or usefulness of any tooth in the maxillary bone. I have passed small sticks through the two perforations, so that you may plainly see where they enter the sinus. I will pass this about for your inspection.

Most works on surgery recommend the use of a trocar or gouge for this purpose, which seems to me is unnecessarily rough and primitive. To be sure, surgeons do not always have a dental engine for this work, nor is it frequently convenient for them to have one at the place and time of operation; consequently it seems to me that this operation is pre-eminently the office of a dental surgeon, for we always have the instruments at hand by which the work can be done quickly and dextrously; and there is no reason why we, as a profession, should not be prepared in every way to treat these cases successfully. I have a case in mind in which a specialist chiselled for one hour in the canine fossa, attempting to enter the antrum. The patient was obliged to be dismissed at the end of this time, in consequence of exhaustion, and the opening had to be made at a subsequent sitting, which still required considerable additional time. With a dental engine this perforation undoubtedly could have been made in much shorter time, probably not occupying over a minute or two, or possibly less after the dissection of the soft tissue had been made, so that I urge upon you as dental surgeons the necessity of our taking up and doing this class of work, not only for the comfort of patients, but for our own benefit as practitioners and surgeons.

I will not go into the medication of these cases, for our essayist has done this in an admirable manner, and besides this the symptoms of the various cases will be obliged to be taken into consideration, and the treatment given accordingly; but I trust the excellent manner in which the essayist has presented this subject will bring it forcibly before you, and that you may in the future treat such patients yourselves instead of sending them to other surgeons.

